# Recent Advances in Androgen Receptor Pathway Inhibitors for Castration-Sensitive Prostate Cancer

**DOI:** 10.3390/ph18111697

**Published:** 2025-11-08

**Authors:** Andrea Lancia, Marco Oderda, Federico Camilli, Eleonora Festa, Marta Bottero, Emanuele Alì, Salvatore La Mattina, Elisabetta Bonzano, Jessica Saddi, Beatrice Detti, David Alberto Santos Hernandez, Gianluca Ingrosso

**Affiliations:** 1 Radiation Oncology, Fondazione IRCCS Policlinico San Matteo, 27100 Pavia, Italy; s.lamattina@smatteo.pv.it (S.L.M.);; 2 Division of Urology, Department of Surgical Sciences, Molinette Hospital, University of Turin, 10124 Turin, Italy; marco.oderda@unito.it; 3 Radiation Oncology Section, Department of Medicine and Surgery, University of Perugia and Perugia General Hospital, 06156 Perugia, Italy; federico.camilli@icloud.com (F.C.);; 4 Radiotherapy Unit, IRCCS Regina Elena National Cancer Institute, 00144 Rome, Italy; 5 Radiation Oncology Unit, Azienda Unitaria Sanitaria Locale-Istituto di Ricovero e Cura a Carattere Scientifico, 42123 Reggio Emilia, Italy; 6 Radiotherapy Unit Prato, Usl Centro Toscana, Presidio Villa Fiorita, 59100 Prato, Italy; 7 Department of Radiation Oncology, Centro Nacional de Radioterapia, San Salvador 1101, El Salvador; 8 Department of Medical Research, Instituto Nacional de Salud, San Salvador 1101, El Salvador

**Keywords:** prostate cancer, androgen receptor pathway inhibitor, surgery, radiotherapy, metastases

## Abstract

Prostate cancer (PCa) is the second most common cancer in men, and it is frequently diagnosed at an advanced stage of the disease. Androgen Deprivation Therapy (ADT) has traditionally represented the backbone of therapy for high-risk, recurrent, and metastatic disease; however, in the last ten years a new group of molecules known as androgen receptor pathway inhibitors (ARPIs) have been demonstrated to improve outcomes in metastatic patients when added to ADT. Developed and validated originally in the setting of castration-resistant disease, ARPIs have been implemented progressively earlier in the natural history of PCa, involving patients who have never received ADT before or that are still responsive to this treatment. Considering the strong evidence for treatment intensification in patients with high-risk features, with this review we aim to provide a complete overview of the current indications for the use of ARPIs through all the stages of castration-sensitive prostate cancer (CSPC).

## 1. Introduction

Prostate cancer (PCa) is the second most common cancer and the fifth leading cause of cancer death in men, globally [1]. The natural history of this disease and its underlying biology, largely driven by testosterone which plays a key role in tumor growth and progression as initially demonstrated by Huggins in 1940 [2], can be extremely heterogeneous, ranging from an indolent state to a very aggressive phenotype, leading to a fatal outcome. Patients affected by localized PCa with aggressive features derive clinical benefits from curative approaches such as surgery or radiation therapy (RT), with or without Androgen Deprivation Therapy (ADT), and in patients with high-risk localized disease, ADT is currently used in combination with radiotherapy to the prostate [3]; however, almost 30–40% of these patients eventually undergo treatment failure [4], with biochemical recurrence often anticipating a macroscopic disease relapse or progression, which can be detected with currently available molecular imaging modalities. Gonadotropin-Releasing Hormone (Gn-RH) agonists and antagonists have been extensively employed as a pharmacological method of ADT in the treatment of PCa, thanks to their ability to induce castration levels of serum testosterone (20–50 ng/dl). Furthermore, ADT serves as the backbone for managing both biochemical recurrence—especially in patients with rapid PSA kinetics—and properly metastatic disease. During biochemical recurrence and in metastatic disease developed after primary treatment, the castration-sensitive phase (PCa that responds to ADT, reflecting its dependence on circulating testosterone) has a median duration of two to five years, after which patients become castration-resistant (biochemical, radiological or clinical progression despite serum testosterone < 50 ng/dl). It has been shown that the time interval between ADT start and the onset of castration resistance is associated with overall survival (OS) [5]. New molecules, such as androgen receptor pathway inhibitors (ARPIs), provide a benefit in terms of survival outcomes and delay the onset of castration resistance, as demonstrated by several landmark trials [6,7]. In the present review, we sought to provide a detailed analysis of the role of ARPIs in castration-sensitive PCa, from localized to metastatic disease.

### 1.1. ARPI Molecular Features and Mechanism of Action

**Figure 1:**
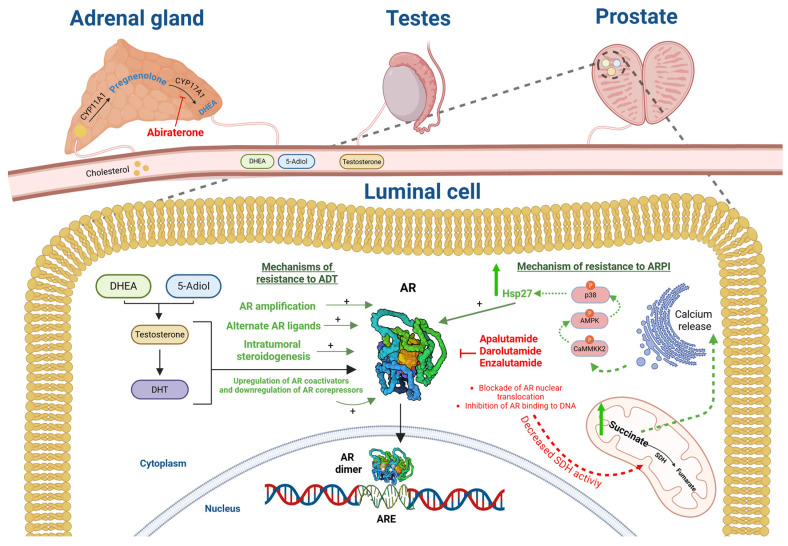
**Mechanism of action of ARPIs and mechanisms of resistance to ADT and ARPIs. DHEA:** Dehydroepiandrosterone; **5-Adiol:** 5-androstenediol; **DHT:** Dihydrotestosterone; **AR:** androgen receptor; **ARE:** androgen response element; **SDH:** succinate dehydrogenase. Created in BioRende SSO, Santos hernández, D. (2025) https://BioRender.com/7qieasn. URL accessed on 10 October 2025.

ARPIs represent a significant advancement in the treatment of PCa, particularly in the context of metastatic disease. A thorough understanding of their molecular features and mechanisms of action is therefore essential for optimizing their use and addressing resistance to therapy. ARPIs are non-steroidal drugs designed to inhibit the androgen receptor (AR), a key driver of PCa progression. Abiraterone acetate, apalutamide, darolutamide, and enzalutamide are included in this category. Abiraterone acetate is converted in vivo to abiraterone, an androgen biosynthesis inhibitor that inhibits 17α-hydroxylase/C17,20-lyase (CYP17), which is required for androgen biosynthesis [8]. Apalutamide, darolutamide, and enzalutamide function by binding to AR’s ligand-binding domain (LBD), blocking androgen translocation to the nucleus and preventing androgen-mediated transcription of target genes involved in tumor growth and survival (Figure 1). The structural composition of the AR is crucial for the mechanism of action of ARPIs. This receptor is made up of three different domains: the N-terminal domain (NTD), responsible for transcriptional activation; the DNA-binding domain (DBD), which allows interaction with specific DNA sequences; and the LBD, which binds androgens [8]. The primary mechanism by which ARPIs exert their therapeutic effects involves competitive inhibition of androgen binding to the AR. This blockade prevents the receptor from activating genes that facilitate cancer cell growth. Furthermore, ARPIs can act by inducing adaptive pathways that support the reactivation of the AR and the onset of castration resistance. For instance, they induce an acute suppression of succinate dehydrogenase (SDH) activity, a fundamental enzyme in the tricarboxylic acid (TCA) cycle. This suppression causes succinate accumulation, which activates intracellular signaling pathways that can stabilize AR protein levels [9]. Specifically, succinate induces calcium release from internal stores, leading to activation of the p-CAMKK2/p-AMPK/p-p38 signaling axis. This cascade enhances levels of Hsp27, an AR co-chaperone that promotes AR stability and activity [9]. Therefore, while ARPIs initially inhibit tumor growth, they can also trigger adaptive responses that foster resistance (Figure 1). Moreover, as PCa progresses under ARPI treatment, it can undergo trans-differentiation into neuroendocrine prostate cancer (NEPCa), a biological entity characterized by low or absent AR expression and aggressive behavior. This transformation is driven by genetic alterations such as gains in MYCN and AURKA oncogenes and losses in tumor suppressor genes like TP53 and RB1 [10]. Such changes lead to increased expression of lineage plasticity genes like SOX2 and EZH2, facilitating a shift towards a more aggressive tumor phenotype [10].

### 1.2. ARPI in Localized Prostate Cancer: A Radiation Oncologist’s Perspective

The introduction of ARPIs has profoundly changed and impacted the treatment of PCa, both in metastatic and non-metastatic disease. In the last few years, several phase III trials have proven that treatment intensification, with the addition of ARPI to ADT, can improve outcomes in the metastatic hormone-sensitive setting [11,12,13,14,15,16]. There is now growing clinical evidence of the benefit of the application of these intensification strategies in the earlier setting of the disease, such as high-risk (HR) PCa and biochemical recurrent PCa. Two to three years of ADT has been the backbone therapy, alongside curative RT, in the setting of HR PCa; nevertheless, recent data suggests that a doublet systemic therapy (ARPI + ADT) leads to improved outcomes; therefore, this new approach is changing clinical practice in these patients (Table 1) [17].

Attard and colleagues [18] demonstrated in 1974 non-metastatic HR patients (cN1M0 or cN0M0 with two HR features: ≥cT3, ISUP ≥ 4, and PSA ≥ 40 ng/mL) within the STAMPEDE platform study that the addition of 2 years of abiraterone to 3 years of ADT improved metastasis-free survival (MFS) (6-year MFS 82%, 95% CI: 79–85 in the combination-therapy group and 69% 95%CI: 66–72 in the control group) and OS (6-year OS 86% in the combination-therapy groups vs. 77% in the control group) with respect to ADT alone. They suggest that this treatment option should represent the standard treatment in this setting. The trial also had a third arm in which patients underwent both abiraterone and enzalutamide, but no benefits in MFS were reported (HR 1.02, 95%CI: 0.70–1.50, *p* = 0.91) at the cost of higher toxicity.

These results are supported by international clinical guidelines. The ESTRO-ACROP consensus guidelines on ADT [23] confirmed that according to STAMPEDE results, 3 years of ADT plus 2 years of abiraterone are recommended for patients with two high-risk features alongside curative RT. Accordingly, the recently published results of the Advanced Prostate Cancer Consensus Conference (APCCC) in 2024 [24] support the STAMPEDE results and its application for HR localized and locally advanced PCa (with 68% of the panelists voting in favor). However, they also conclude that due to the lack of data on the survival outcomes of other ARPI (such as darolutamide, enzalutamide or apalutamide) in this setting, their use is not yet deemed suitable.

Several ongoing studies are evaluating the role of ARPI in the HR PCa curative setting: the ENZARAD (ANZUP 1303) trial [NCT02446444], the DASL-HiCaP trial [NCT04136353], and ATLAS [NCT02531516].

Moreover, genomic tests (such as Decipher) have been implemented in ongoing clinical trials to define which patients may derive the greatest benefit from the use of a more intensified treatment strategy with ARPI. The PREDICT-RT [NCT04513717] is an ongoing phase III trial that randomizes HR PCa patients based on Decipher score. Patients with a score < 0.85 were eligible for a de-intensified approach, RT + 12 months of ADT or RT + 24 months of ADT, while patients with a Decipher score > 0.85 were enrolled in the intensified arms: RT + 24 months of ADT or RT + 24 months of ADT + 24 months of apalutamide. The primary endpoint of the study is whether apalutamide can improve MFS for patients with a high Decipher score, and whether those with a lower score can be treated with a less intensified strategies and achieve comparable MFS rates.

As for the HR setting, much has changed lately in the management of biochemical recurrent PCa. While salvage RT (sRT) remains the only curative treatment option for patients experiencing biochemical recurrence (BCR) after radical prostatectomy, intensified treatment strategies with the addition of ARPI have been proposed for patients with HR features, some of them even without the use of RT.

The Embark trial [19] has revolutionized the clinical practice in BCR patients, changing the paradigm of ADT alone as the cornerstone of systemic treatment for BCR patients. More specifically, this phase III trial published by Freedland and colleagues showed that enzalutamide alone or associated with ADT improves MFS in patients with BCR and HR feature (such as PSA doubling time ≤ 9 months and PSA ≥ 2 ng/mL above nadir after RT or ≥1 ng/mL after RP). Five-year MFS was 87.3% with ADT + enzalutamide vs. 71.4% with ADT alone, and 80.0% with enzalutamide monotherapy.

Moreover, regarding intensified treatment strategies for BCR patients, several prospective phase II trials have been published, randomizing patients with HR features to receive sRT with intensified systemic treatment. The SALV-ENZA trial [20] randomized patients to sRT + placebo vs. sRT + 6 months of enzalutamide, reporting a 2-year freedom form PSA progression of 66% and 84% respectively.

In the FORMULA-509 trial [21], 345 patients were treated with sRT + 6 months ADT and randomized to Bicalutamide vs. Abiraterone + apalutamide. Three-year progression-free survival (PFS) was 46.8% for Bicalutamide vs. 67.2% for the abiraterone and apalutamide group. Finally, the RTOG 3506-STEEL trial [22] randomized patients to RT + 24 months of ADT +/− enzalutamide, and PFS was in favor of arm 2 but at a cost of higher toxicity rates.

Multiple prospective trials are ongoing, and following the important results of the EMBARK trial, ARPI will likely become a crucial part of the treatment paradigm in the setting of HR BCR patients, hopefully maintaining sRT as a combination strategy.

In conclusion, the evolving evidence supporting the combination of ARPI and ADT, particularly in HR localized PCa and HR BCR patients, underscores a significant shift in clinical practice, with ongoing trials aimed at further refining patient selection for treatment intensification strategies.

### 1.3. ARPI in Localized Prostate Cancer: A Surgeon Perspective

Approximately 22% of new diagnosed PCa cases are classified as HR, a life-threatening condition that requires an aggressive and often multimodal treatment [25,26]. Nevertheless, in patients undergoing radical prostatectomy the use of neoadjuvant hormonal treatments is currently not recommended, based on historical data coming from trials on luteinizing hormone-releasing hormone (LHRH) agonists and first-generation antiandrogens that failed to show survival benefits [27,28].

A renewed interest toward the adoption of neoadjuvant treatments has arisen with the introduction in the clinical practice of the androgen receptor pathway inhibitors (ARPIs) that demonstrated dramatic improvement in survival outcomes in the metastatic setting. In the last 10 years, nine studies evaluated the use of ARPIs before prostatectomy in high- and intermediate-risk patients, alone or in different combinations. Of these, all were phase II clinical trials, most of them randomized controlled trials (RCT), and three conducted as single-arm studies (Table 2) Promising results were reported utilizing pathological complete response (pCR, intended as absence of residual tumor at histological examination, up to 14% [29]) and minimal residual disease (MRD, defined as residual tumor burden ≤ 5 mm at cross-sectional slice, up to 38% [30]) as pathological endpoints. The rates of pCR + MRD were higher in studies evaluating neoadjuvant apalutamide (38% [30]) and darolutamide (40% [31]), as compared to abiraterone and enzalutamide (up to 30% when used in combination [32]). Survival outcomes were reported in only three studies [31,33,34]. In the first of these, Efstathiou et al. showed that recurrence-free survival (RFS) at 3 years was 75% in patients receiving abiraterone plus leuprolide vs. 71% in those receiving leuprolide alone [33]. In the second study, the NEAR trial, a single-arm study in which 30 patients received a 12-week course of neoadjuvant apalutamide before prostatectomy the biochemical RFS was 86% at 2 years in [34]. In a later study, Zhuang et al. reported the results of 30 patients treated with 6 months of ADT plus darolutamide prior to surgery, where a 1-yr PFS of 90% was observed [31]. A strong correlation between pathological responses and biochemical RFS was demonstrated by McKay et al., with only two cases of biochemical recurrence and no metastases in patients with a pathological response after almost 4 years of follow-up [35]. Two RCTs evaluated the combination of different ARPIs, abiraterone plus enzalutamide [32] or abiraterone plus apalutamide [36] in a super-intensification protocol, without demonstrating a significant benefit in pCR or MRD as compared to an ARPI alone. In all the aforementioned studies, the neoadjuvant treatment with ARPI lasted for a duration that ranged from 3 to 6 months.

The ARPIs could also be administered after radical prostatectomy in an adjuvant setting (Table 1), even though current European guidelines discourage using any kind of ADT in pN0 patients, irrespectively of the risk class, and consider standard ADT in pN1 patients only, without any ARPI [27]. In this setting, two trials evaluated the benefits of ARPIs as adjuvant treatments, both investigating apalutamide, alone [37] or in combination with abiraterone [38]. In 2023, McKay et al. showed that treatment with apalutamide, abiraterone, prednisone, plus leuprolide for 12 months resulted in a non-statistically significant improvement in 3-year biochemical RFS for patients receiving treatment [38]. More recently, Shore et al. reported in a single-arm series of 108 men with high-risk localized PCa treated with 12-months adjuvant ADT + apalutamide a very promising biochemical RFS at 2 years of 100% with good tolerability [37].

Although ARPIs in combination with surgery in a neoadjuvant or adjuvant setting is not recommended, we are waiting for the results of several phase III trials that might change the current guidelines. Notably, the PROTEUS (NCT03767244) and STAPLE trials (NCT06758882) are both evaluating apalutamide + ADT in neoadjuvant and/or adjuvant settings, further expanding the role of surgery in a multimodal approach. The PROTEUS trial randomizes patients with very-high-risk PCa to ADT plus apalutamide or placebo 6 months before and after prostatectomy, while the STAPLE trial investigates the addition of apalutamide to ADT following prostatectomy in oligometastatic patients in prostate-specific membrane antigen positron emission tomography.

**Table 2:** Studies evaluating the use of ARPIs in the adjuvant and neoadjuvant settings for PCa.

Author, Year	Therapy	Design	Arms of Neoadjuvant	No of Patients	Positive Margins	pCR	MRD	pCR + MRD	Survival
	**NEOADJUVANT TREATMENTS**								
Taplin, 2014 [39]	ABI	Phase II RCT, 58 high-risk men	A: 12-w ABI + L vs. B: 24-w ABI + L	A: 28 pts B: 30 pts	19% vs. 10% (p NR)	4% vs. 10% (p NR)	0% vs. 14% (p NR)	4% vs. 24% (p NR)	-
Montgomery, 2017 [40]	ENZA	Phase II RCT, 52 intermediate/high-risk men	A: 6-mo ENZA vs. B: 6-mo ENZA + dutasteride + L	A: 27 pts B: 25 pts	16% vs. 22% (p NR)	0% vs. 4% (p NR)	0% vs. 13% (p NR)	0% vs. 17% (p NR)	-
Efstathiou, 2019 [33]	ABI	Phase II RCT, 65 high-risk men	A: 3-mo L vs. B: 3-mo ABI + L	A: 21 pts B: 44 pts	14% vs. 5% (p 0.17)	-	-	-	71% vs. 75% 3-yr RFS (p 0.28)
McKay, 2019 [32]	ABI + ENZA	Phase II RCT, 75 intermediate/high-risk men	A: 24-w ENZA + L vs. B: 24-w ABI + ENZA + L	A: 25 pts B: 50 pts	12% vs. 18% (p NR)	8% vs. 10% (p NR)	8% vs. 20% (p NR)	16% vs. 30% (p 0.2)	-
McKay, 2021 [36]	ABI + APA	Phase II RCT, 118 intermediate/high-risk men	A: 6-mo ABI + L vs. B: 6-mo ABI + APA + L	A: 59 pts B: 59 pts	12% vs. 7% (p NR)	10% vs. 13% (p NR)	10% vs. 9% (p NR)	20% vs. 22% (p 0.4)	-
Lee, 2022 (NEAR trial) [34]	APA	Phase II single-arm, 30 intermediate/high-risk men	12-w APA	30 pts	16%	0%	NR	NR	86% 2-yr bRFS
Devos, 2023 (ARNEO trial) [30]	APA+ Degarelix	Phase II RCT, 89 high-risk men	A: 3-mo Degarelix + APA vs. B: 3-mo Degarelix + placebo	A: 45 pts B: 44 pts	18% vs. 18% (p NR)	0% vs. 0% (p NR)	38% vs. 9% (p 0.002)	38% vs. 9% (p 0.002)	-
Wei, 2023 [29]	APA	Phase II single-arm, 7 advanced PCa	4-mo APA + ADT	7 pts	-	14%	-	-	-
Zhuang, 2024 [31]	DARO	Phase II single-arm, 30 high-risk men	6-mo DARO + ADT	30 pts	13%	7%	33%	40%	90% 1-yr PFS
	**ADJUVANT TREATMENTS**								
McKay, 2023 [38]	ABI + APA	Phase II RCT, 82 IR/HR men	A: 12-mo ABI + APA + L vs. B: observation	A: 42 pts B: 40 pts	14 (17%)	4-yr: 67% vs. 61%	-	-	-
Shore, 2024 (NCT04523207) [37]	APA	Phase II single-arm, 108 HR men	12-mo APA + ADT	108 pts	6 (6%)	2-yr 100%	-	-	-

**ABI:** abiraterone; **ENZA:** enzalutamide; **DARO:** darolutamide; **APA:** apalutamide; **pCR:** pathological complete response; **MRD:** minimal residual disease.

### 1.4. ARPI in De Novo Metastatic Castration-Sensitive Prostate Cancer

De novo metastatic castration-sensitive prostate cancer (mCSPC) represents approximately 5–10% of all PCa diagnoses, and it is associated with nearly 50% of PCa-related deaths [41,42].

It typically shows more aggressive behavior and a shorter time to castration resistance than metachronous mCSPC. ADT has been the cornerstone treatment for mCSPC, but despite initial responses, most patients eventually progress to castration-resistant prostate cancer (CRPC). This disease progression mainly occurs because of continued AR signaling, often due to mutations or alterations in AR or related pathways. However during the last 10 years, several large randomized clinical trials have demonstrated how the addition of ARPIs to ADT has improved patient outcomes in de novo mCSPC (Table 3) [11,12,13,14,15,16]. Significant improvements were observed in OS, PFS, and radiographic progression-free survival (rPFS), particularly in cases of high-volume symptomatic disease and good performance status patients; these achievements translated into the adoption of personalized treatment approaches in the clinics, with an acceptable safety profile, even though these trials stratified patients according to clinical criteria not necessarily reflecting the biology of the specific therapy [41].

The first study that demonstrated the benefit of an upfront combination therapy with ARPI was the double-blinded phase III trial LATITUDE, which reported an OS advantage in patients with de novo high-risk mCSPC treated with abiraterone acetate and ADT compared to those treated with ADT alone, with a median OS of 53.3 months and 36.5 months, respectively, without having identified predictive biomarkers of response to abiraterone acetate [11].

There are currently no strong indications as to which ARPI to favor in the setting of mCSPC; overall, treatment choice can be reasonably guided by patients’ comorbidities, side effects, need of concomitant medications or duration of therapy. Two phase III clinical trials, the ARCHES and the ENZAMET trials [13,14], evaluated the role of enzalutamide with ADT, with a benefit in terms of a 61% risk reduction in radiographic disease progression compared with ADT alone in the first study and a 5-year OS of 67% in the enzalutamide group compared with 57% in the control group (non-steroidal first-generation anti-androgen like Bicalutamide or flutamide) in the latter study, with an advantage in terms of OS in all prognostic subgroups with respect to disease burden and time of onset of metastases. It should be underlined that in the ARCHES trial, approximately 36% of the patients had low-volume disease, about 25% had received prior local therapy, and 18% of the patients had been treated with prior docetaxel [14]. However, in the final prespecified analysis, OS was significantly improved with a decrease of the risk of death by 34% compared with ADT alone (HR 0.66, 95%CI:0.53–0.81, *p* < 0.001).

At the same time, the efficacy of apalutamide in the de novo mCSPC setting has also been reported. The double-blinded phase III trial TITAN [15], which included more than 80% of patients with metastatic synchronous disease, showed a reduction in the risk of death by 35% with apalutamide combined with ADT compared to ADT alone (HR: 0.65, 95%CI: 0.53–0.79, *p* < 0.0001). After the initial unblinding, there was a cross-over of approximately 40% of the patients in the control group to the experimental arm, and this resulted in a 48% reduction in the risk of death. Nearly 62.7% of patients had high-volume disease, and in the subgroup analysis the benefit from apalutamide was reported in almost all subgroups.

Unlike previously reported studies, a correlation between clinical outcome and biomarkers was investigated in the TITAN trial, reporting a strong association between the detection of circulating tumoral DNA (ctDNA) or AR genomic alterations and a poor OS in both treatment groups (both biomarkers measured at baseline and at the end of the study) [15,43].

Across the major international guidelines, the role of ARPIs in de novo mCSPC is consistently highlighted, proving a significant shift in the treatment paradigm where it has become part of the new standard of care, especially for high-risk patients [44].

Several attempts have been made to find strategies to further intensify systemic treatment in mCSPC patients; for instance, with the addition of chemotherapy. While the CHAARTED trial and STAMPEDE trial demonstrated that adding docetaxel to ADT in high-volume metastatic disease improved OS and PFS compared to ADT alone [12,45]. Another two phase III trials, ARASENS and PEACE-1, investigated the role of triplet therapy (docetaxel, ADT, and ARPI) as treatment intensification, showing that this strategy improves survival outcomes with an acceptable safety profile with particular regard to high-volume symptomatic patients [16,46]. ARASENS enrolled 1306 patients, 86.1% of them with de novo mCSPC, and showed a 32.5% lower risk of death (HR: 0.68, 95%CI: 0.57–0.80, *p*< 0.001) in the group with triplet therapy (darolutamide, docetaxel, and ADT) compared to the one receiving placebo [16]. In a later analysis, the OS benefit was reported mainly in the high-volume (HR: 0.69, 95%CI: 0.57–0.82) and high-risk (HR: 0.62, 95%CI: 0.42–0.90) patients, who are the most-represented subgroups in the trial, 77% and 70%, respectively [47].

At the same time, the PEACE-1 trial [46], enrolling 1173 patients with de novo mCSPC, showed that the addition of abiraterone to ADT, docetaxel, and RT to the primary tumor significantly reduced the relative risk of radiographic progression by 46% (4.46 yrs vs. 2.22 yrs, HR: 0.54, 95%CI: 0.41–0.71) and, after a median follow-up of almost 5 years, was associated with an 18% reduction in the risk of death (5.72 yrs vs. 4.72 yrs, HR: 0.82, 95%CI: 0.69–0.98, *p* = 0.03) compared to those who did not receive it, and these results were more pronounced in patients with high-volume disease.

Triplet therapy is therefore an important option for the management of high-risk or high-volume de novo mCSPC, especially as exploiting the cytotoxic effects of docetaxel and the androgen receptor inhibition of ARPIs can improve clinical outcome as reported above. However, the decision to use triplet therapy requires careful consideration of various clinical factors and must be tailored according to comorbidities, patients’ life expectancy, disease burden, and performance status.

**Table 3:** Studies evaluating the use of ARPIs in metastatic castration-sensitive prostate cancer.

Author, Year	Therapy	Design	Arms of Treatment	No of Patients	OS	PSAP	rPFS	CRFS
Fizazi, 2017 (LATITUDE trial) [11]	ABI	Phase III RCT, 1119 high-risk mCSPC men	A: ADT B: ADT + ABI	A: 602 pts B: 597 pts	mOS: 36.5 months vs. 53.3 months	mPSAP 7.4 months vs. 33.2 months	mrPFS: 14.8 months vs. 33 months	__
Armstrong, 2019 (ARCHES trial) [14]	ENZA	Phase III RCT, 1150 de novo or with recurrence mCSPC men	A: ADT B: ADT + ENZA	A: 574 pts B: 576 pts	Immature data HR for death 0.81 *p* = 0.33 for group B	HR 0.19 *p* < 0.001 for group B	mrPFS: 19 months vs. NR	Median time to castration resistance: 13 months vs. NR
Davis, 2019 (ENZAMET) [13]	ENZA	Phase III RCT, 1125 mCSPC men	A:ADT B: ADT + ENZA	A: 562 pts B: 563 pts	72% Vs. 80% 3-yr OS	67% Vs. 37% 3-yr PSAPFS	__	__
Chi, 2019 (TITAN trial) [15]	APA	Phase III RCT, 1051 mCSPC men	A: ADT B: ADT + APA	A: 527 pts B: 525 pts	73.5% vs. 82.4% 2-yr OS	mPSAP12.9 months vs. NR	mrPFS 22.1months vs. NR	__
Smith, 2022 (ARASENS trial) [16]	DARO	Phase III RCT, 1306 mCSPC men	A: ADT + DOCE B: ADT + DOCE + DARO	A: 651 B: 655	mOS 48.9 months vs. NR	__	__	Median time to castration resistance: 19.1 months vs. NR
Fizazi, 2022 (PEACE-1 trial) [46]	SOC vs. SOC + ABI	Phase III RCT, 1172 de novo mCSPC men	A: ADT or DOCE B: ADT or DOCE + RT C: ADT or DOCE + ABI D: ADT or DOCE + RT + ABI	Group I A: 296 + B: 293 Group II C: 292 + D: 291	mOS 4.7 years vs. 5.7 years	__	mrPFS 2.2 years vs. 4.5 years	mCRPS 1.5 years vs. 3.8 years
Davis, 2022 (sub-analysis ENZAMET trial) [48]	ENZA	Phase III RCT, 270 high-volume mCSPC men	A: ADT + DOCE B: ADT + ENZA + DOCE	A: 137 B: 133	__	HR OS: 0.79 (0.57–1.10) 5-yr OS	__	__

**ABI:** abiraterone; **ENZA:** enzalutamide; **DARO:** darolutamide; **APA:** apalutamide; **OS**: overall survival; **PSAP:** PSA progression; **rPFS:** radiographic progression-free survival; **CRFS:** castration-resistant-free survival.

### 1.5. ARPI in Metachronous Castration Sensitive Prostate-Cancer

Patients with metachronous mCSPCare, by definition, are those who typically show distant relapse after having presented with localized disease for which they were initially offered definitive therapy. The distinction between metachronous and synchronous (i.e., de novo) presentation is critical, as patients in these two groups have different prognoses, influencing the subsequent treatment choice. More specifically, metachronous metastatic disease has been associated with a better prognosis.

As previously mentioned, ADT has been the cornerstone of treatment in metastatic PCa, and multiple therapeutic strategies including new generation hormone therapies have been validated and currently represent the standard of care approach for the management of patients with mCSPC. In several pivotal clinical trials, the addition of ARPIs to standard ADT has demonstrated significant benefits in terms of PFS and OS [11,12,13,14,15,16]. For metachronous mCSPC patients, in whom ADT alone must be considered an undertreatment, ARPIs offer a valuable tool for delaying further disease progression. Furthermore, the addition of ARPIs has been associated with improved quality of life due to a reduction in disease-related symptoms.

The biology of metachronous CSPC may significantly diverge from that of synchronous CSPC in terms of different molecular pathways, resistance mechanisms, genomic mutations, and androgen receptor signaling profiles; this makes treatment more complex in this setting but also provides an opportunity to exploit residual sensitivity to AR inhibition and discriminate cases for treatment intensification [49,50].

These two groups—synchronous and metachronous—can be further categorized according to the extent of metastatic disease at diagnosis as low- and high-volume CSCP patients, following the CHARTEED criteria (presence of visceral metastases or at least four bone lesions, with one or more lesions located beyond the vertebral bodies and pelvis) [45]. Therefore, we are able to recognize four distinct subgroups of metastatic CSPC patients: synchronous high-volume CSPC, synchronous low-volume CSPC, metachronous high-volume and metachronous low-volume CSPC. While we currently have data showing that treatment intensification, for instance, adopting triplet regimens with or without primary radiotherapy, can improve survival outcomes in metastatic PCa [46,47], it is important to note that specifically in the setting of metachronous CSPC disease, such an approach may represent an overtreatment in a significant proportion of these patients. Additionally, its potential toxicity—both from a physiological and financial perspective—needs to be carefully considered.

Makarov et al. [51] analyzed a series of 3096 patients who underwent radical prostatectomy. Of these, 422 developed biochemical recurrence (PSA > 0.2 ng/mL) and were managed with surveillance until time of metastasis, which eventually occurred in 123 patients; 41 died of prostate cancer. The median time between PSA failure and metastasis development was 32 months, and median time from metastasis to prostate cancer mortality was 82 months. These findings suggest that patients affected by mCSPC can be very different and the natural history of mCSPC can vary; therefore, the role of duplet/triplet therapy is crucial, especially for the management of high-risk or high-volume de novo mCSPC, while the treatment of metachronous mCSPC should deserve a different treatment paradigm. Eight-year OS CHAARTED data presented by Tripathi et al. [52] demonstrates that docetaxel added to testosterone suppression shows clear OS benefit in synchronous high-volume mCSPC and in metachronous high-volume mCSPC; meanwhile, modest effects in synchronous low-volume disease and no meaningful effects in metachronous low-volume disease were reported (HR: 0.77; 95% CI: 0.51 to 1.18), inferring that both the volume and timing of metastatic disease are prognostic and predictive for the benefit of docetaxel.

The same does not hold true for treatment with second-generation anti-androgens. Updated OS data from ENZAMET demonstrated that enzalutamide added to testosterone suppression improves PFS and OS across all four subgroups [48]. The subgroup analysis from this study, which allowed the use of docetaxel, reveals that adding docetaxel to enzalutamide and testosterone suppression provides a survival benefit only in the group with the worst prognosis (i.e., synchronous, high-volume). These results align with those observed in PEACE-1 [46] and ARASENS [16].

It has been hypothesized that different biology may influence the clinical phenotypes associated with metachronous or synchronous mCSPC; in this scenario, clinical variables for prognosis and treatment selection for the different patients’ subgroups will need to be addressed. Hamid et al. published results from Dana-Farber’s mCSPC cohort in 2019 and showed that patients without p53, RB1, or PTEN mutations had much longer OS rates [53]. Data from the John Hopkins University cohort by Sutera et al. published in 2022 also showed that patients without p53 mutations and oligometastatic disease had the best rPFS rates [54].

Beyond intensified systemic therapy, evidence has been collected on the role of metastasis-directed therapy (MDT) in selected patients with metachronous, oligometastatic mCSPC. A systematic review and meta-analysis of 22 prospective studies of MDT in patients with metastatic PCa demonstrated that the estimated 2-year PFS0 was 46%, with 2-year local control, ADT-free survival, and OS of 97%, 55%, and 97% respectively. MDT is also associated with a favorable safety profile, with grade 2 and grade ≥ 3 adverse events occurring in 2.4% and 0.3% of patients, respectively [55]. In addition to clinical and pathological factors, genomic profiling seems to offer prognostic value in the MDT context. A combined analysis of 70 prostate cancer patients with genomic data from two major trials assessing the role of MDT in oligometastatic prostate cancer patients showed that patients with high-risk mutations (ATM, BRCA1/2, RB1, or TP53) experienced the greatest benefit in PFS from MDT (HR for high-risk = 0.05; HR for no high-risk = 0.42; *p*-value for interaction = 0.12) [56]. Within the MDT group, the PFS was 13.4 months for patients without a high-risk mutation, compared to 7.5 months for those with a high-risk mutation (HR: 0.53; 95% CI: 0.25–1.11; *p* = 0.09).

One of the key advantages of ARPIs’ use in metachronous mCSPC is their ability to specifically target the AR signaling axis without inducing the systemic side effects seen with traditional chemotherapy. The mechanism of action is highly effective in patients with residual sensitivity to androgens, as it is often the case with metachronous disease.

### 1.6. Network Map Analysis of ARPIs Use Across Different Disease Stages

**Figure 2:**
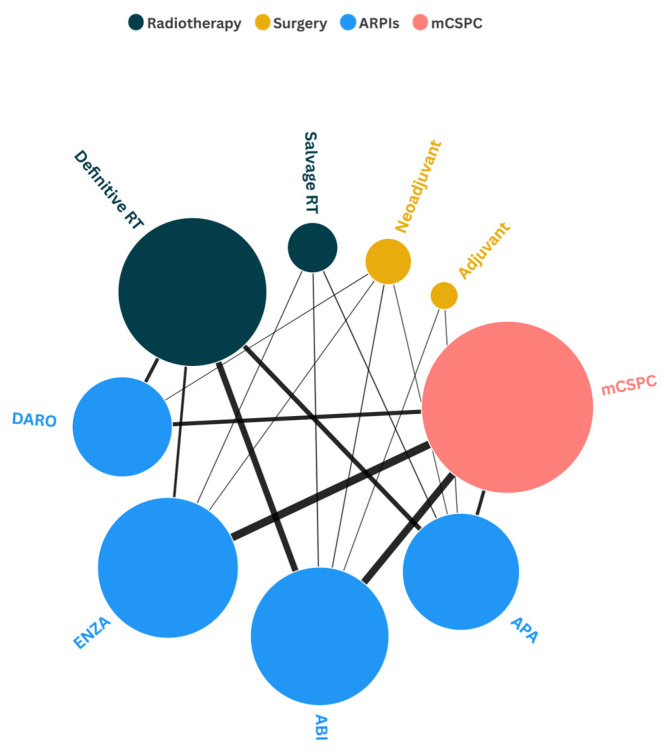
**Network graph of ARPIs integration in prostate cancer.** The size of each circle represents the total number of patients enrolled in studies evaluating each intervention. The thickness of the lines reflects the total number of patients included in studies assessing combination of interventions. **DARO:** darolutamide; **ENZA:** enzalutamide; **ABI:** abiraterone; **APA:** apalutamide; **mCSPC:** metastatic castration-sensitive prostate cancer; **RT:** radiation therapy.

The ARPIs have been most widely investigated in the mCSPC setting and in combination with radical RT (Figure 2).

Enzalutamide and abiraterone are the most extensively studied ARPIs, with phase III clinical trial evidence supporting their use in the metastatic setting (mCSPC). Additional phase III data from the STAMPEDE trial [18] supports the use of abiraterone in combination with radical RT in localized high-risk patients. Both agents have also been investigated in combination with sRT in phase II clinical trials [19,20,21,22].

Regarding the newer ARPIs, darolutamide and apalutamide, these agents have already been evaluated in phase III clinical trials for mCSPC [15,16]. Ongoing phase III clinical studies are further evaluating their use in combination with radical RT; meanwhile, only apalutamide has been investigated to date in salvage settings with RT [21].

In the surgical setting, abiraterone has been the most frequently investigated, mainly as a neoadjuvant treatment, with evidence available from phase II clinical trials, followed by apalutamide, for which a phase III trial is currently ongoing.

## 2. Discussion and Conclusions

PCa represents one of the leading causes of cancer death in men, and according to the Lancet Oncology commission, new cases are expected to rise by the end of 2040, with a burst of diagnoses of the more advanced stages of the disease [57].

ARPIs represent the breakthrough of prostate cancer treatment and have revolutionized outcomes during the last decades. They show a major affinity for the AR than the older non-steroidal antiandrogen molecules previously developed and extensively used in the clinics during the last decades.

Developed and validated originally in the setting of castration resistant-disease, the use of ARPIs has been implemented progressively earlier in the natural history of prostate cancer, involving patients who have never received ADT before or that are still responsive to this treatment as noted in Figure 2. There is currently strong evidence for treatment intensification in mCSPC, where ADT alone does not represent the standard of care anymore.

Several landmark trials [11,12,13,14,15,16] have shown how ARPIs have found their new sweet spot in this stage of disease, leading to improved survival, excellent tolerability when added to ADT, and delayed time to castration resistance. The ARASENS, ARCHES, and PEACE-1 trials reported a 64–71% reduction in the risk of progression to castration resistance with ARPI treatment (35% of events in the darolutamide group vs. 60% in the placebo group in the ARASENS trial) and a prolongation of the time to castration resistance, as shown in Table 3, with no variation in the incidence of adverse events (AEs) compared with the control groups.

However, despite these data, real world scenarios are different; it has been hypothesized that almost two-thirds of mHSPC patients are still receiving ADT only. Several reasons may explain this undertreatment rates; it may be due to unjustifiable safety concerns regarding their use or limited access to the drugs, related to their higher overall treatment cost, but in the majority of cases there may be an underestimation of the clinical benefit of the molecules or the will to postpone their use in the castration-resistant disease for which they were originally validated (Figure 2), since the use of ARPI’s in mCSPC has been shown to be cost-effective in the United States and Canada [58,59]. However, in Europe, although an increase in quality-adjusted life years has been observed, cost-effectiveness has not yet been established, which may be attributed to heterogeneity of economic data and the limited availability of real-world evidence, as most economics estimates are derived from pivotal clinical trials [60].

No head-to-head comparisons have been made until now, so the choice is largely dependent on the occurrence rates of adverse event profiles or physician preference. Each molecule presents a characteristic AEprofile which may partially overlap with the ones of the other sister drugs; therefore, the selection should be individualized according to patient comorbidities and expected tolerance. Abiraterone, due to its mineralocorticoid effect, should be avoided in patients with cardiovascular disease (heart failure, uncontrolled hypertension), liver dysfunction or diabetes, comorbidities that are common in older adults. Enzalutamide and apalutamide, on the other hand, can be used under these conditions. However, due to their ability to cross the blood–brain barrier, they may cause central nervous system adverse effects (fatigue, cognitive disturbances); thus, they should be avoided in frail or elderly patients and those with seizure risk. Darolutamide, in turn, exhibits the most favorable safety profile and can be used in the aforementioned patient population, as well as in those with polypharmacy.

As a general rule, understanding the molecular features and mechanisms of action of the different ARPIs is essential for improving therapeutic outcomes in PCa. By targeting the AR signaling pathways while recognizing potential adaptive responses, clinicians can develop more effective treatment strategies. The emergence of resistance mechanisms highlights the need for combination therapies that target multiple pathways. For example, co-inhibition strategies involving poly (ADP-ribose) polymerase inhibitors and ARPIs have been explored in the castration-resistant setting, whereas data on the combination of them are accumulating [61].

On the other side, the EMBARK trial [19] has opened the doors for the use of ARPI in the non-metastatic hormone-naive patients, namely those undergoing biochemical recurrence and presenting with high-risk features based on their histology, prostate-specific antigen doubling time, and genomics; an advantage in term of MFS was observed in those patients presenting high-risk BCR and no evidence of macroscopic disease on conventional imaging who received either ENZA +ADT or ENZA alone, compared to those who received ADT only.

To conclude, in the extremely heterogeneous scenario of advanced prostate cancer, we believe it will be crucial to identify biomarkers that predict response to ARPIs and guide personalized treatment approaches.

Ongoing trials are investigating optimal sequencing and combination strategies, which could further enhance the therapeutic potential of ARPIs. Moreover, understanding the molecular underpinnings of metachronous prostate cancer could help identify which patients are most likely to benefit from ARPI therapy and tailor treatments accordingly.

## Figures and Tables

**Table 1:** Studies evaluating the use of ARPIs in combination with RT in different settings or alone for the treatment of biochemical recurrence.

Author, Year	Therapy	Design	Arms of Treatment	No of Patients	MFS	OS	FFP	PFS
DEFINITIVE TREATMENT								
Attard, 2022 (STAMPEDE platform) [18]	RT + ABI	Phase III RCT, 1974 high-risk men	A: RT + ADT alone B: RT + ADT + ABI	A: 988 pts B: 986 pts	82% vs. 69% vs. 6-yr MFS	86% vs. 77% vs. 6-yr OS	__	__
NCT02446444 (ENZARAD trial) ongoing	RT + ENZA	Phase III RCT, 802 non-metastatic high-risk or N1	A: RT + ENZA B: RT + ADT	802 pts	__	ongoing	ongoing	__
NCT04136353 (DASL-HiCaP trial) ongoing	RT + DARO	Phase III RCT, 1100 non metastatic high-risk men	A: RT + ADT + DARO B: RT + ADT	110 pts	ongoing	ongoing	ongoing	__
NCT02531516 (ATLAS trial) ongoing	RT + APA	Phase III RCT, 1503 non metastatic high-risk men	A: RT + APA + ADT B: RT + ADT	1503 pts	ongoing	ongoing	ongoing	ongoing
**SALVAGE TREATMENT**								
Freedland, 2023 (EMBARK trial) [19]	ENZA	Phase III RCT, 1068 high-risk men with biochemical recurrence	A: ENZA + ADT B: ADT alone C: ENZA alone	A: 355 pts B: 358 pts C: 355 pts	87.3% vs. 71.4% vs. 80% 5-yr MFS	92% vs. 87.2% vs. 89.5% 5-yr OS	__	__
Tran, 2022 (SALV-ENZA trial) [20]	SRT + ENZA	Phase II RCT, 86 high-risk men with biochemical recurrence	A: SRT + ENZA B: SRT alone	A: 43 pts B:43 pts	__	__	66% vs. 84% 2-yr FFP	__
Nguyen, 2023 (FORMULA-509) [21]	SRT + ABI + APA	RCT, 345 high-risk men with biochemical recurrence	A: SRT + ADT6m + BI B: SRT + ADT6m + ABI + APA	A: 172 B: 173	A: 66% vs. B: 84% 3-yr MFS	__	__	69% vs. 75% 3-yr PFS
Posadas, 2024 (STEEL trial) [22]	SRT + ADT + ENZA	Phase II RCT, 188 high-risk men with biochemical recurrence	A: SRT + ADT B: SRT + ENZA	A: 94 pts B: 94 pts	__	__	__	HR PFS for arm B: 0.72 one side *p* = 0.14

RT: radiotherapy; ABI: abiraterone; ENZA: enzalutamide; DARO: darolutamide; APA: apalutamide; SRT: salvage radiotherapy; MFS: metastasis-free survival; OS: overall survival; FFP: freedom from progression; PFS: progression-free survival; pts: patients; yr: years.

## Data Availability

No new data were created or analyzed in this study. Data sharing is not applicable to this article.
